# Rhyming and Mnemonic Key to Materia Medica

**Published:** 1891-09

**Authors:** 


*Rhyming and Mnemonic Key to Materia Medica.* By M.D. Pp. 48. London: L. & J. PARNELL. [n.d.]

If this booklet, to which the author has not had the courage to attach either his name or the date, is not intended as a burlesque, it is about the most stupid production we have ever seen. Its purpose is sufficiently indicated by its title, but the rhyme is of the feeblest and the mnemonics are more puzzling than the actual details of the Pharmacopœia. We almost owe an apology to our readers for quoting any of the drivel which is here offered as an aid to the student's memory. The following is a sample of that which is to be found running through the whole book:

> "And then note you well, Troch. Morph. et Ipecacu.
> Is made with Morph., Ipecac., and Tinct. of Tolu;
> Morph. hydro. 1–36th and 1-12th Ipec. in each loz.,
> Give this for a cough if trouble it cause."

We trust that before long all copies of this work will have disappeared, so that the next generation may not think that the medical education of to-day could be associated with such twaddle. Possibly some members of an overstocked profession might welcome it, as its use would be likely to prevent students passing their examinations.

